# Real-World Effectiveness of Elexacaftor/Tezacaftor/Ivacaftor in Cystic Fibrosis: A 24-Month Italian National Registry Study

**DOI:** 10.3390/jcm15072699

**Published:** 2026-04-02

**Authors:** Donatello Salvatore, Giuseppe Campagna, Rita Padoan, Angela Pepe, Annalisa Amato, Marco Salvatore

**Affiliations:** 1 Italian Cystic Fibrosis Patients League ODV-LIFC, 00162 Rome, Italy; giuscampagna@gmail.com (G.C.); ritaf54@gmail.com (R.P.); amatoann@libero.it (A.A.); 2 Faculty of Medicine and Psychology, La Sapienza University, Medical-Surgical Sciences and Translational Medicine, 00161 Rome, Italy; 3 Italian Cystic Fibrosis Registry, Higher Institute of Health, 00161 Rome, Italy; marco.salvatore@iss.it; 4 Cystic Fibrosis Center, Department of Maternal and Child Health, Hospital San Carlo, 85100 Potenza, Italy; angpepe01@gmail.com; 5 Undiagnosed Rare Diseases Interdepartmental Unit, National Center Rare Diseases, Higher Institute of Health, 00161 Rome, Italy

**Keywords:** cystic fibrosis, registry, elexacaftor/tezacaftor/ivacaftor

## Abstract

**Background:** The CFTR modulator elexacaftor/tezacaftor/ivacaftor (ETI) has transformed cystic fibrosis (CF) care, but national-level real-world data on long-term effectiveness, durability of response, and treatment de-escalation remain limited. **Methods:** We conducted a nationwide longitudinal study using the Italian Cystic Fibrosis Registry. People with CF aged ≥6 years who initiated ETI between October 2019 and December 2022 and received ≥3 months of continuous therapy were included. Lung function (percent predicted FEV_1_, ppFEV_1_), nutritional status (BMI or BMI z-score), hospital days, complications, microbiology, and chronic treatments were assessed during the two years before and up to two years after ETI initiation. Longitudinal changes were analyzed using generalized estimating equations with multiple imputation for missing data. **Results:** The cohort included 2276 individuals (mean age 27.9 ± 13.3 years; 49% female). Mean ppFEV_1_ declined during the pre-ETI period but increased by 9.9 percentage points at 12 months after ETI initiation (*p* < 0.001) and remained 6.8 percentage points above baseline at 24 months. A decline between 12 and 24 months was observed overall, except in individuals with severe baseline lung disease (ppFEV_1_ < 40%), who maintained stable improvements. Mean annual hospital days decreased by approximately 65% and remained low throughout follow-up. Nutritional status improved, with a mean BMI increase of approximately 1.05 kg/m^2^ compared with immediate pre-treatment in adults and a BMI z-score increase of 0.2 SD compared with pre-treatment timepoints in children. Use of most standard CF therapies declined substantially, particularly among individuals with ppFEV_1_ ≥ 40%. The prevalence of allergic bronchopulmonary aspergillosis decreased, while liver disease prevalence increased modestly, largely reflecting transient elevations in liver enzymes. **Conclusions:** In this nationwide real-world cohort, ETI was associated with sustained improvements in lung function, nutritional status, and hospitalization burden. The attenuation of lung function gains after the first year, particularly in less severe disease, supports the need for individualized monitoring and cautious treatment de-escalation in the ETI era.

## 1. Introduction

Cystic fibrosis (CF) is a life-limiting autosomal recessive disorder caused by variants in the cystic fibrosis transmembrane conductance regulator (CFTR) gene, affecting approximately 170,000 individuals worldwide. Progressive lung disease, driven by impaired mucociliary clearance, chronic infection, and persistent inflammation, remains the leading cause of morbidity and mortality. While early diagnosis through newborn screening and advances in symptom management have extended median survival, the burden of progressive lung damage remains substantial [1,2].

The introduction of CFTR modulators has fundamentally transformed CF care. These small molecules target the underlying molecular defect by improving CFTR protein folding, trafficking, or channel gating. The triple combination elexacaftor/tezacaftor/ivacaftor (ETI), approved in 2019, represents the most significant advance to date [3]. ETI combines two correctors with a potentiator to address F508del-CFTR dysfunction, the most common mutation, present in approximately 70% of people with CF (pwCF) globally.

Pivotal trials demonstrated unprecedented efficacy, with mean improvements of 10–14 percentage points in percent predicted FEV_1_ (ppFEV_1_), marked reductions in pulmonary exacerbations (PEx), and significant quality of life gains [4,5]. These findings led to regulatory approval for individuals aged ≥2 years carrying at least one F508del allele or another responsive variant. Real-world studies have largely confirmed these benefits, demonstrating sustained improvements in lung function, reduced hospitalization rates, and enhanced nutritional outcomes [6,7,8,9]. However, considerable interindividual variability in treatment response has been observed, potentially influenced by genotype, baseline disease severity, prior modulator exposure, age, and comorbidities [10,11].

Italy hosts one of Europe’s most comprehensive national CF registries. The Italian CF Registry (ICFR) collects longitudinal data from all accredited CF centers, enabling evaluation of outcomes across heterogeneous patient groups [12]. Following regulatory approval, ETI became available through the Italian National Health Service for individuals aged ≥2 years carrying at least one F508del allele, providing an opportunity for systematic assessment of its real-world impact.

This study uses ICFR data to provide nationwide, real-world evidence of ETI effectiveness in an unselected population of pwCF aged ≥6 years. By comparing lung function and nutritional trajectories during the 24 months before and after ETI initiation, we aimed to quantify treatment benefits and examine responses across clinically relevant subgroups. Our findings will help inform evidence-based management strategies in the era of highly effective CFTR modulation.

## 2. Materials and Methods

The present study uses data from the ICFR, which includes all pwCF in Italy (estimated coverage 97%). In 2022, 6077 pwCF were included in the registry [12].

In Italy, ETI has been in use since October 2019, when the first few pwCF initiated treatment as part of a compassionate-use programme. ETI became commercially available in Italy for all pwCF aged ≥12 years with at least one copy of the F508del variant in July 2021. ETI subsequently became available for pwCF aged 6–11 years in September 2022. Although ETI reimbursement in Italy is regulated by Italian Drug Agency (AIFA) eligibility criteria, the registry includes individuals who received ETI through other authorized pathways (e.g., compassionate use, clinical trials, early access, or special funding). In addition, patients listed for lung transplantation were not systematically excluded, as national regulatory practice allows CFTR modulator therapy in selected cases, with documented removal from the transplant waiting list following clinical improvement.

This study population includes all pwCF who initiated ETI at one of the twenty-seven CF centres and services in Italy between 21 October 2019 and 31 December 2022 and who had ≥3 months of continuous ETI treatment. During follow-up, some individuals discontinued ETI or died; follow-up was censored at treatment discontinuation (absence of ETI for ≥3 months), death, or end of the study period (31 December 2024), whichever occurred first. Lung transplantation was not observed among ETI-treated individuals.

ICFR collects data on lung function and nutrition follow-up, recording the value of FEV_1_ in litres of the highest ppFEV_1_ of the year; the height measured at the date of best FEV_1_; and the weight measured at the date of best FEV_1_.

The reported spirometry values are converted to percentage of predicted values according to Global Lung Initiative reference equations [13]. Height and weight are converted to Body Mass Index (BMI) (weight in kilograms divided by the square of the height, kg/m^2^) for subjects older than 18 years old, and to BMI z-score calculated according to the CDC reference values for children (younger than 18 years old) [14].

Because data were recorded only once per year, lung function and nutritional parameters collected in the same year as ETI initiation (Time 0, T0) were excluded from the analysis to avoid capturing measurements during the acute phase of improvement following treatment initiation. The observation period included a 24-month lookback window to assess pre-ETI trends preceding T0 (T − 24 and T − 12) and ended 24 months after ETI initiation (T + 12 and T + 24), death, treatment discontinuation, or the end of the study period, whichever occurred first.

Data on complications (allergic bronchopulmonary aspergillosis (ABPA), CF-related diabetes (CFRD), liver disease (LD), pneumothorax, massive haemoptysis, and malignancy), lung microbiology, and chronic medications (inhaled hypertonic saline, inhaled RhDNase, inhaled antibiotics, inhaled bronchodilators (BD), oxygen, azithromycin (AZM), ursodeoxycholic acid (UDCA), and pancreatic enzyme replacement therapy), and total number of days spent in hospital during the year were recorded for the year preceding and the two years following initiation of ETI. Liver disease (LD) was categorised as LD without cirrhosis or LD with cirrhosis, with the latter including cirrhosis with or without portal hypertension.

The favourable opinion for the activities carried out within the ICFR was obtained in 2020 and renewed by the National Ethics Committee for Research Involving Public Research Bodies and other national public entities (CEN) on 17 April 2023 (Istituto Superiore di Sanità protocol no. AOO-ISS-27/04/2023-0019953, Class: PRE BIO CE 01.00). All pwCF or their legal representatives provided written, informed consent to have their data included in the ICFR. This study on anonymous patient data was approved by the ICFR Scientific and Steering Committees on 18 February 2025 (N. 02/2025). The terms of use of the provided data are governed by Italian law in accordance with the European data protection legislation (GDPR EU Regulation 2016/679 and Legislative Decree of 30 June 2003, No. 196, as amended by Legislative Decree of 10 August 2018, No. 101 Provisions for the adaptation of national legislation to the provisions of EU Regulation 2016/679–GDPR).

The assessed CF disease progression and treatment effectiveness outcomes were: (i) lung function, measured by ppFEV_1_, with pulmonary function tests available only in individuals aged ≥6 years in accordance with Global Lung Function Initiative standards; (ii) presence of the clinically relevant bacterial pathogen *Pseudomonas aeruginosa*; and (iii) body mass index (BMI) in adults and BMI z-score in individuals younger than 18 years.

CF-related complications and comorbidities were evaluated as safety outcomes, including the prevalence of ABPA, CFRD, physician-reported hepatic events, and the incidence of pneumothorax, massive haemoptysis (>250 mL blood loss per day), and malignancy.

For chronic therapies and hospitalizations, we compared the prevalence of pwCF receiving inhaled and oral treatments, long-term oxygen therapy, and the total number of hospital days in the pre-ETI and ETI periods; death and lung transplantation were also evaluated.

### Statistical Analysis

Continuous variables are presented as mean ± standard deviation (SD) and 95%CI (Confidence Interval), while categorical variables are shown as absolute frequency and percentage, n (%).

In the longitudinal analysis of the variables BMI, BMI z-score, and ppFEV_1_, there was a mean percentage of missing data between 6% and 10% (imputation is recommended as a rule of thumb when missing data is greater than 5%), therefore we used imputation techniques to address the missing data problem and to avoid so-called listwise deletions that lead to unreliable results. Simply eliminating patients with missing data would reduce the power of the study and lead to an underestimation of the variability, and hence would narrow the confidence interval. Imputation was obtained using “proc mi” and “proc mianalyze”, routines in the SAS software, with a number of imputations ranging from 5 to 25 and with a non-monotonic data distribution [15,16,17,18].

Longitudinal data analysis was performed through GEE (Generalized Estimating Equations), in which we assessed both the difference over time and the difference between group * time interaction.

McNemar’s test was used to evaluate the difference between two proportions with respect to the dependent categorical variables with two modalities. Absence and presence of liver disease with and without cirrhosis were evaluated by the Bowker test (omnibus), and McNemar’s test was used as post hoc analysis.

Statistical analysis was performed using software SAS version 9.4 TS Level 1 M8 and JMP PRO v. 17.1 (SAS Institute, Cary, NC, USA).

A *p*-value < 0.05 was considered statistically detectable.

## 3. Results

### 3.1. Study Population

A total of 2276 pwCF who initiated ETI in Italy between 21 October 2019 and 31 December 2022 were included. The mean (SD) age at treatment initiation was 27.93 (13.29) years. Females accounted for 48.99% of the cohort; 35.76% had the F508del (F)/F genotype, and 64.24% had an F/other genotype. Adults represented 73.46% of the cohort (Table 1).

### 3.2. Lung Function Outcomes

#### 3.2.1. ppFEV_1_ and Days of Hospitalization

**Figure 1:**
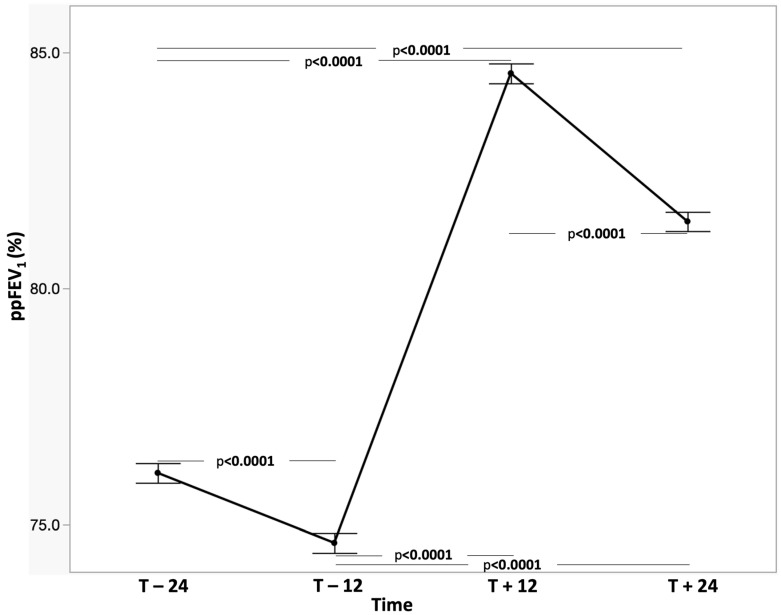
Plot of ppFEV_1_ at different times (overall population).

At two years before ETI initiation (T − 24), mean (SD) ppFEV_1_ was 76.09 (25.29) (95% Confidence Interval (CI): 75.04 to 77.15). Lung function declined during the pre-treatment period, reaching 74.63 ± 25.45% (95% CI: 73.57 to 75.69) one year before initiation (mean difference: 1.46 percentage points; 95% CI: 1.05 to 1.88; *p* < 0.0001). Following treatment initiation, ppFEV_1_ increased to 84.56 ± 25.62 (95% CI: 83.50 to 85.62) at 12 months, corresponding to a mean improvement of 9.93 percentage points compared with the immediate pre-treatment value (95% CI: 9.44 to 10.41; *p* < 0.0001). At 24 months post-initiation (T + 24), ppFEV_1_ was 81.42 ± 24.80 (95% CI: 80.39 to 82.46), representing a sustained improvement of 6.79 percentage points relative to the immediate pre-treatment period (95% CI: 6.28 to 7.31; *p* < 0.0001). However, ppFEV_1_ declined significantly between 12 and 24 months (mean difference: 3.13 percentage points; 95% CI: 2.67 to 3.59; *p* < 0.0001) (Table 2 and Figure 1).

Although PEx data were not systematically captured by ICFR during the study period, total hospital days per year served as a surrogate measure of exacerbations requiring inpatient management. Mean annual hospital days decreased substantially following ETI initiation, from a mean (SD) of 7.56 (15.63) days at two years pre-treatment (T − 12) to 2.61 ± 9.70 days at one-year post-treatment (T + 12) and 2.49 ± 7.62 days at two years post-treatment (T + 24). This represents a mean reduction of 4.95 days per year (95% CI: 4.26 to 5.63; *p* < 0.0001) at T + 12 versus T − 12 and 5.07 days per year (95% CI: 4.42 to 5.72; *p* < 0.0001) at T + 24 versus T − 12, corresponding to approximately 65% fewer hospital days. The reductions were sustained through both treatment years with no evidence of increase between T + 12 and T + 24 (mean difference: 0.12 days; 95% CI: −0.31 to 0.55).

#### 3.2.2. Genotype Subgroups

Improvements in lung function were observed across genotypes. In individuals with the F/F genotype, mean ppFEV_1_ increased from 76.02 ± 26.00 (95% CI: 74.21–77.83) at baseline to 85.70 ± 25.80 (95% CI: 83.91–87.49) at 12 months (T − 12). Similarly, in individuals with an F/other genotype, ppFEV_1_ increased from 73.84 ± 25.19 (95% CI: 72.53–75.15) to 83.90 ± 25.54 (95% CI: 82.59–85.22) at T + 12. Between 12 and 24 months (T + 12 to T + 24), both groups experienced modest declines in ppFEV_1_ (F/F: 82.69 ± 24.80%; 95% CI: 80.96–84.42; F/other: 80.72 ± 24.78%; 95% CI: 79.44–82.00) (Table 3).

To further characterize the F/other subgroup, patients were stratified according to the functional class of the second CFTR variant into F/gating (F/G), F/residual function (F/RF), and F/minimal function (F/MF). In the F/G subgroup, ppFEV_1_ increased at T + 12 from 67.94 ± 28.31 (95% CI: 58.52–77.36) to 73.72 ± 29.61 (95% CI: 64.30–83.14), followed by a return to baseline values at T + 24 (67.90 ± 27.94%; 95% CI: 58.96–76.84). The F/RF subgroup also showed an improvement in ppFEV_1_ at T + 12 compared with baseline (from 79.61 ± 21.98%; 95% CI: 75.79–83.43 to 85.93 ± 22.33%; 95% CI: 82.12–89.74), with a subsequent decline at T + 24 (81.53 ± 22.14%; 95% CI: 77.70–85.37). Finally, individuals with an F/MF genotype demonstrated a marked improvement at T + 12 (from 73.45 ± 25.33%; 95% CI: 71.71–75.19 to 84.62 ± 25.14%; 95% CI: 82.90–86.33), followed by only a minimal decline at T + 24 (82.11 ± 24.45%; 95% CI: 80.43–83.79). All changes from T − 12 to T + 12 and from T + 12 to T + 24 were statistically significant (*p* < 0.0001) (Table S1).

#### 3.2.3. Clinical Subgroups

No significant differences in ppFEV_1_ were observed between females and males (Table 4). In contrast, paediatric patients showed significantly higher ppFEV_1_ values than adults (Table 5). Similarly, individuals without CFRD (Table 6) and those without *P. aeruginosa* infection (Table 7) had significantly better lung function than those with these conditions. Despite these baseline differences, the longitudinal pattern of ppFEV_1_ was consistent across all subgroups: a significant improvement from 12 months before ETI initiation (T − 12) to 12 months after initiation (T + 12), followed by a decline between T + 12 and T + 24, mirroring the trend observed in the overall cohort.

Individuals with mild-to-moderate lung disease (ppFEV_1_ ≥ 40%) exhibited trends similar to those observed in the overall cohort, whereas those with severely impaired lung function (ppFEV_1_ < 40%) showed a sustained improvement over time. In the latter group, mean ppFEV_1_ increased from 31.38 ± 7.70% (95% CI: 30.00–32.70) at baseline to 37.33 ± 10.07% (95% CI: 35.69–38.97) at 12 months, with this gain maintained at 24 months (37.98 ± 8.85%; 95% CI: 36.51–39.46) (Table 8).

To further characterise the mild-to-moderate group, individuals were stratified into moderate (ppFEV_1_ ≥ 40% and <70%) and mild (ppFEV_1_ ≥ 70%) lung disease. Both subgroups showed a similar pattern, with improvement during the first year of ETI treatment followed by a decline in the second year; however, the different magnitude of this decline was clinically relevant. In the moderate lung disease subgroup, ppFEV_1_ increased from 51.78 ± 11.77 (95% CI: 50.79–52.76) at T − 12 to 61.99 ± 13.64 (95% CI: 60.87–63.11) at T + 12, and then slightly declined at T + 24 (60.06 ± 13.65%; 95% CI: 58.93–61.19). In contrast, the mild lung disease subgroup showed a substantial increase from T − 12 to T + 12, from ppFEV_1_ 89.07 ± 17.14 (95% CI: 88.18–89.96) to 99.26 ± 15.59 (95% CI: 98.45–100.06), followed by a more pronounced decline between T + 12 and T + 24 (95.10 ± 16.51%; 95% CI: 94.24–95.96) (Table S2).

### 3.3. Nutritional Outcomes

Mean BMI at two years before initiation was 21.67 ± 2.99 kg/m^2^ (95% CI: 21.53–21.82) and increased to 21.82 ± 3.04 kg/m^2^ (95% CI: 21.67 to 21.96) one year before initiation. Following treatment initiation, BMI rose to 22.87 ± 3.15 kg/m^2^ (95% CI: 22.72 to 23.02) at 12 months, an increase of 1.05 kg/m^2^ compared with immediate pre-treatment (95% CI: 0.97–1.14; *p* < 0.0001) and 1.20 kg/m^2^ compared with two years pre-treatment (95% CI: 1.11–1.28; *p* < 0.0001). The distribution widens post-therapy, with more patients achieving higher BMIs. At 24 months, BMI remained stable (22.93 ± 3.25 kg/m^2^; 95% CI: 22.72 to 23.02), with no significant change from 12 months (mean difference: 0.06 kg/m^2^; 95% CI: −0.12 to 0.008).

**Figure 2:**
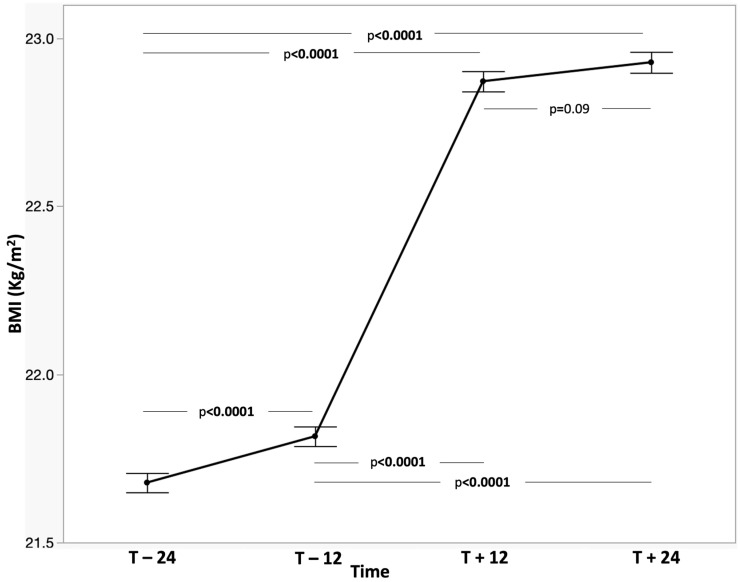
Plot of BMI at different times (overall population).

**Figure 3:**
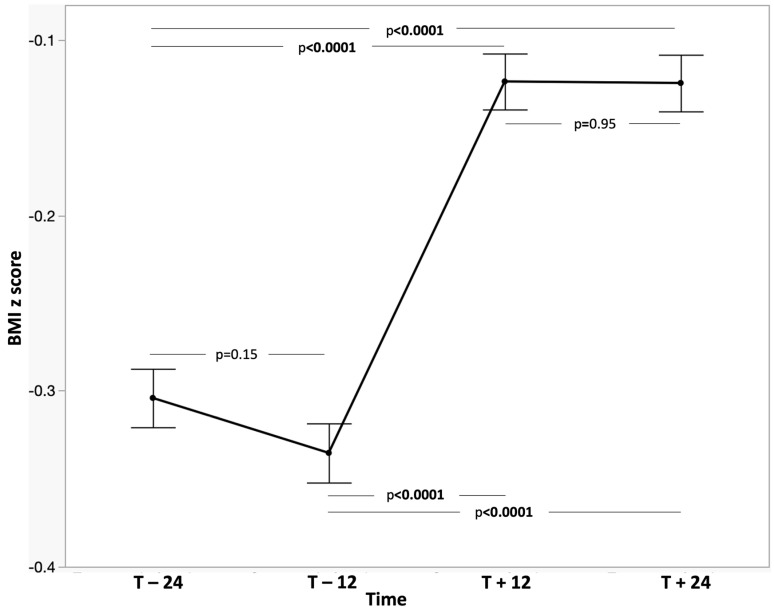
Plot of BMI z-score at different times (overall population).

Among paediatric participants, mean BMI z-score was −0.30 ± 1.04 (95% CI: −0.39 to −0.22) two years before initiation, and remained unchanged one year before (mean difference: 0.04; 95% CI: −0.01 to 0.07). After treatment initiation, BMI z-score improved to −0.12 ± 1.00 (95% CI: −0.20 to −0.04) at 12 months, an increase of 0.21 SD compared with both pre-treatment timepoints (95% CI: 0.16–0.26; *p* < 0.0001). This improvement was sustained at 24 months (−0.12 ± 1.01; 95% CI: −0.20 to −0.04) with no significant change from 12 months (Table 2). The visualization shows minimal visible change in distribution shape (Figure 2 and Figure 3). The overall trends were similar in all the subgroups.

### 3.4. Complications and Comorbidities

Prevalence of chronic *P. aeruginosa* infection fluctuated between one year before and the two years after initiation. Allergic bronchopulmonary aspergillosis (ABPA) decreased progressively from the pre-treatment period through both treatment years. Prevalence of diabetes did not improve and showed a tendency to increase. Pneumothorax remained rare, while episodes of massive haemoptysis declined significantly. Prevalence of malignancy remained stable (Table 9).

Physician-reported hepatopathy was classified as liver disease (LD) with or without cirrhosis. The prevalence of LD without cirrhosis decreased from 37.90% to 26.70% during the first year of treatment and subsequently increased to 49.02% in the second year. In contrast, cirrhotic LD showed only modest fluctuations over time (Table 10).

No evidence of progression from non-cirrhotic to cirrhotic LD was observed during the evaluation period (Time T − 12 vs. T + 12 *p* = 0.67, time T − 12 vs. T + 24 *p* = 0.37, and time T + 12 vs. T + 24 *p* = 0.47).

### 3.5. Treatments

Use of most standard CF therapies declined substantially after ETI initiation. Inhaled hypertonic saline use decreased significantly during the first treatment year and then stabilized. Inhaled RhDNase use also declined markedly during both years following initiation. Inhaled BD use mildly increased in the first two years of ETI treatment. Use of inhaled antibiotics fell sharply across both years. Long-term oxygen therapy decreased significantly during the first year. Regarding oral therapies, pancreatic enzyme replacement therapy use remained stable. Use of UDCA increased, especially between the first and second treatment years. Oral AZM use decreased markedly across both years (Table 9).

To determine whether treatment reduction reflected disease severity, patterns were examined by baseline ppFEV_1_. Among pwCF with severe lung disease (ppFEV_1_ < 40%), hypertonic saline use sharply declined, and RhDNase showed a less marked decrease; use of inhaled BD declined in the first year but recovered in the second, whereas inhaled antibiotics and oxygen therapy remained stable. In contrast, pwCF with ppFEV_1_ ≥ 40% showed significant, progressive reductions across all inhaled standard CF therapies, except for BD. Increased UDCA use was limited to this less severely affected group. Both groups declined significantly in oral AZM (Table 11 and Table 12).

## 4. Discussion

This nationwide analysis of 2276 people with CF (pwCF) initiating ETI demonstrates substantial and sustained clinical benefits through 24 months of treatment, though with an unexpected decline in lung function during the second year. The mean ppFEV_1_ improved by 10.0 percentage points at 12 months and remained 6.8 percentage points above baseline at 24 months, while body mass index (BMI), treatment burden, and hospitalizations all showed durable improvements. These real-world outcomes confirm ETI’s transformative impact on CF care, while highlighting important questions about long-term response patterns and optimal management strategies in the post-modulator era.

Recent Italian real-world studies evaluating the effectiveness of ETI have generally been limited to single-center cohorts or relatively small multicenter populations, typically including approximately 80–200 patients with follow-up ranging from 6 to 24 months. These studies consistently report improvements in lung function, BMI, and reductions in pulmonary exacerbations; however, most focus on specific subgroups, such as individuals with advanced lung disease, paediatric populations, specific genotypes, or patients switching from previous CFTR modulators, and therefore include narrower age ranges and smaller cohorts [10,19,20,21,22]. In contrast, the present study analyses one of the largest real-world Italian cohorts treated with ETI and includes a broader age distribution, providing a more comprehensive evaluation of treatment effectiveness in routine clinical practice. The size and heterogeneity of this population enhance the generalizability of the findings and enable assessment of ETI outcomes across a wider spectrum of disease severity and age groups than previously reported in Italian cohorts.

Our first-year ppFEV_1_ improvement of 10 percentage points aligns closely with pivotal trials [4,5] and international registry data from Denmark (13.0 percentage points; 95% CI: 11.3–14.6), Germany (11.3; 95% CI: 10.8–11.8), the United States (8.2; 95% CI: 8.0–8.4), and the United Kingdom (9.4; 95% CI: 9.0–9.8) [6,9,23,24]. However, our cohort diverged markedly in the second year. While US and UK registries demonstrated continued improvement (Year 2 gains of 8.9 and 10.2 percentage points, respectively), we observed a mean decline of 3.1 percentage points (95% CI: 2.67–3.59) between months 12 and 24, resulting in a net 24-month improvement of 6.8 percentage points (95% CI: 6.28–7.31) [6,9].

This pattern, initial improvement followed by partial decline while remaining above baseline, appeared consistently across genotypes (F508del homozygous and heterozygous), sex, age groups, and presence of chronic *P. aeruginosa* infection or CFRD. A critical exception emerged when stratifying by baseline disease severity. Patients with ppFEV_1_ ≥ 40% followed the overall pattern of Year 2 decline, whereas those with severe impairment (ppFEV_1_ < 40%) maintained their 12-month gains through 24 months. Strikingly, the magnitude of decline between 12 and 24 months correlated inversely with baseline severity: no decline in severe disease, −1.6 percentage points in moderate disease, and −4.2 percentage points in mild disease. This differential response likely reflects ceiling effects in patients with preserved lung function, though differences in treatment adherence [25] or disease trajectory cannot be excluded from registry data.

Genotype-specific analyses revealed nuanced patterns. F508del homozygous patients and those with F508del/gating (F/G) or F508del/residual function (F/RF) mutations, most previously treated with older CFTR modulators, showed comparable improvements to prior studies [6]. The F/G subgroup exhibited return to baseline ppFEV_1_ at 24 months, potentially indicating limited additive benefit from elexacaftor and tezacaftor when CFTR potentiation is already optimized, though the small sample size (n = 38) necessitates cautious interpretation. In contrast, patients with F508del/minimal function (F/MF) genotypes, truly CFTR modulator-naïve, demonstrated marked 12-month improvements with less pronounced subsequent decline, suggesting sustained benefit in this previously untreated population.

The mechanisms underlying the second-year decline remain uncertain. Potential explanations include ongoing disease progression despite CFTR modulation, incomplete correction of the basic defect, reduced adherence to ETI or standard therapies, or methodological limitations of annual assessments that may miss within-year variability. Importantly, the decline did not translate into increased hospitalizations, which remained substantially reduced through 24 months, suggesting either stabilization at a new baseline or a protective effect against severe exacerbations despite attenuated lung function gains.

Nutritional improvements were sustained throughout follow-up. Adult BMI increased by approximately 1 kg/m^2^ from an already normal baseline, consistent with emerging concerns about overweight and obesity in the modulator era [26,27,28]. Paediatric BMI z-scores improved by 0.2 SD (95% CI: 0.08–0.32) and remained stable through 24 months, particularly notable given negative baseline z-scores of Italian children with CF [28]. Patients with severe lung disease showed the greatest nutritional gains, with mean BMI increasing from 20.7 to 22.3 kg/m^2^.

Chronic *P. aeruginosa* infection prevalence fluctuated across the study period, though interpretation is limited by reduced sputum production on ETI and substitution of oropharyngeal swabs, which have higher false-negative rates. Registry studies suggest approximately 40% of chronically infected patients transition to non-chronic status after one year of ETI, with clearance showing an all-or-none pattern across lung regions regardless of local disease severity [29,30]. Allergic bronchopulmonary aspergillosis (ABPA) prevalence decreased progressively, supporting a disease-modifying effect through reduced inflammation and *Aspergillus fumigatus* colonization [31]. Massive haemoptysis episodes declined significantly, consistent with overall disease improvement [32].

CFRD prevalence showed a tendency to increase during follow-up, reflecting the complex and incompletely understood effects of ETI on glucose metabolism. While some studies report transformative benefits, others document persistent β-cell dysfunction [33,34]. These findings, coupled with ETI’s nutritional effects, necessitate re-evaluation of screening and management protocols, particularly as younger patients initiate therapy earlier in life.

Physician-reported liver disease showed fluctuating prevalence, driven mainly by changes in non-cirrhotic disease, while cirrhosis remained stable, and progression was not observed. These patterns likely reflect heterogeneity in diagnostic criteria and registry coding practices, as well as the frequent reporting of transient transaminase elevations during ETI therapy [35,36,37]. Without standardized diagnostic adjudication, conclusions regarding hepatic disease prevalence should be interpreted cautiously.

Use of standard CF therapies declined substantially after ETI initiation, with distinct patterns by baseline severity. Patients with ppFEV_1_ < 40% reduced only hypertonic saline use, while those with ppFEV_1_ ≥ 40% showed broad, progressive reductions across all therapies except UDCA, which increased. These patterns align with European and US registry data documenting sustained decreases in chronic respiratory therapies [38,39,40]. The PROMISE study demonstrated similar trends, with mean therapy use declining from 2.6 to 1.4 treatments by 54 months in participants aged ≥12 years, without differences in ppFEV_1_ or symptoms between those continuing multiple therapies versus those using fewer [41].

However, our severity-stratified analysis raises important considerations. Patients with ppFEV_1_ ≥ 40%, who demonstrated the most aggressive therapy reductions, subsequently experienced lung function decline between years 1 and 2, whereas those with ppFEV_1_ < 40%, who maintained more treatments, particularly inhaled antibiotics, exhibited stable lung function through 24 months. Whether this reflects natural disease progression, consequences of treatment de-escalation, differential adherence, or an interplay of factors cannot be determined from registry data. Nevertheless, the pattern suggests that treatment response may vary by baseline severity and underscores the need for individualized monitoring and de-escalation strategies [42]. The stability observed in severely impaired patients maintaining selective therapies may reflect more cautious, personalized treatment reduction, an approach that appears clinically prudent given their sustained benefits.

Mean annual hospital days declined by 65% (from 7.6 to 2.5 days), a clinically meaningful reduction consistent with previous studies [43,44], and maintained through 24 months without rebound despite the lung function decline. As CF hospitalizations predominantly reflect pulmonary exacerbations requiring intravenous antibiotics, this metric serves as a reasonable surrogate for severe events, though it does not capture outpatient-managed exacerbations. The persistence of hospitalization reductions despite second-year ppFEV_1_ decline suggests either stabilization at a new disease baseline or a protective effect of ETI against acute exacerbations, though changes in clinical practice toward outpatient management may also contribute.

This study has important methodological limitations that constrain the interpretation of our findings. First, our reliance on annual lung function assessments (recording only the best ppFEV_1_ value each year) represents a critical limitation. This approach inadequately captures within-year variability and cannot distinguish whether the observed second-year decline represents gradual deterioration, episodic events following acute exacerbations, or measurement artifact. The timing of annual assessments may have systematically biased results if patients were more likely to be assessed during periods of clinical stability in Year 1 versus periods encompassing acute events in Year 2. More frequent, protocolized spirometry would be essential to clarify this pivotal finding.

Second, the absence of systematic pulmonary exacerbation data constitutes a major gap. While annual hospital days served as a surrogate for severe exacerbations, this metric missed outpatient-managed events entirely. As patients’ baseline health improved on ETI, the proportion of exacerbations managed without hospitalization likely increased substantially, rendering our hospitalization-based inferences incomplete. Without comprehensive exacerbation data, we cannot definitively assess whether the Year 2 ppFEV_1_ decline reflected increased exacerbation frequency, inadequate exacerbation treatment, or other factors.

Third, the absence of an untreated control group fundamentally limits causal inference. We cannot distinguish treatment effects from natural disease evolution, regression to the mean, or secular trends in CF care quality. The differential patterns observed across severity subgroups are associative, not causal, and alternative explanations, including differences in disease trajectory, treatment practices, or unmeasured confounders, cannot be excluded.

Additional limitations constrain specific outcome interpretations. Changes in sputum production following ETI initiation reduced respiratory culture sampling and increased reliance on oropharyngeal swabs, which have substantially higher false-negative rates. This ascertainment bias complicates the interpretation of *P. aeruginosa* prevalence trends. Registry coding of ‘liver disease’ likely reflects mild transaminitis (elevated alanine aminotransferase and aspartate aminotransferase up to 5 times the upper limit of normal), a known ETI effect [35,36,37], rather than clinically significant hepatic injury or liver failure, precluding definitive hepatic safety conclusions without standardized diagnostic criteria and systematic liver function monitoring. Sweat chloride measurements had excessive missing data and could not be reliably assessed. The registry does not capture respiratory physiotherapy use, adherence to ETI or standard therapies, or patient-reported outcomes regarding treatment burden and quality of life, critical gaps given the substantial treatment regimen changes observed. The absence of detailed adherence data particularly constrains our ability to determine whether the observed lung function decline relates to reduced medication-taking behaviour.

These methodological constraints, particularly the reliance on annual ‘best’ spirometry values, missing comprehensive exacerbation and adherence data, microbiological ascertainment bias, and absence of a control group, substantially limit definitive conclusions about long-term effectiveness, safety, and optimal management strategies. Our findings should be interpreted as hypothesis-generating observations requiring confirmation through prospective studies with frequent protocolized assessments, comprehensive outcome capture, and appropriate control groups.

## 5. Conclusions

This real-world analysis confirms ETI’s transformative impact on CF care, with sustained improvements in lung function, nutrition, and clinical stability through 24 months. The unexpected second-year decline in ppFEV_1_, observed selectively in patients with better baseline lung function, represents a critical finding requiring further investigation. The differential patterns across severity subgroups, coupled with contrasting treatment reduction strategies, raise important questions about the relationship between therapy de-escalation and long-term outcomes. The emergence of newer CFTR modulators with potentially different long-term response profiles, such as vanzacaftor/tezacaftor/deutivacaftor, which offer the potential advantage of once-daily administration and may improve treatment adherence, underscores the need for continued surveillance of real-world effectiveness and safety as the therapeutic landscape evolves.

Our findings underscore that while ETI enables meaningful reductions in treatment burden for many patients, decisions regarding standard therapy continuation should be individualized based on disease severity, ongoing clinical response, and shared decision-making. Future research should focus on identifying predictors of sustained response, optimal timing and pace of therapy de-escalation across severity groups, and mechanisms underlying the observed decline in less severely affected patients. Prospective studies with standardized frequent assessments, systematic adherence monitoring, detailed exacerbation capture, and patient-reported outcomes are essential to guide evidence-based personalized care as the CF population increasingly lives with highly effective CFTR modulator therapy.

## Figures and Tables

**Table 1:** Demographic and clinical characteristics with respect to the reference year.

Parameter	n (%)
Sex	
Male	1161 (51.01)
Female	1115 (48.99)
Patient	
Paediatric	604 (26.54)
Adult	1672 (73.46)
ppFEV_1_ (%)	
<40	151 (6.93)
≥40	2029 (93.07)
Missing	96 (4.2)
*Pseudomonas aeruginosa*	
No	1025 (45.19)
Yes	1243 (54.81)
Missing	8 (0.35)
Diabetes	
No	1635 (75.52)
Yes	530 (24.48)
Missing	111 (4.88)
Genotype	
F/F	814 (35.76)
F/Other	1462 (64.24)

**Table 2:** Overall nutrition, ppFEV_1_ and days of hospitalization.

Parameter	T − 24 Mean ± SD (95% CI)	T − 12 Mean ± SD (95% CI)	T + 12 Mean ± SD (95% CI)	T + 24 Mean ± SD (95% CI)
Hospitalization (days) (n = 2276)	4.52 ± 10.22 (4.08 to 4.96)	7.56 ± 15.63 (6.91 to 8.21)	2.61 ± 9.70 (2.21 to 3.02)	2.49 ± 7.62 (2.17 to 2.81)
BMI (kg/m^2^) (n = 1672)	21.67 ± 2.99 (21.53 to 21.82)	21.82 ± 3.04 (21.67 to 21.96)	22.87 ± 3.15 (22.72 to 23.02)	22.93 ± 3.25 (22.77 to 23.09)
BMI z-score (n = 605)	−0.30 ± 1.04 (−0.39 to −0.22)	−0.34 ± 1.06 (−0.42 to −0.25)	−0.12 ± 1.00 (−0.20 to −0.04)	−0.12 ± 1.01 (−0.20 to −0.04)
ppFEV_1_ (%) (n = 2276)	76.09 ± 25.29 (75.04 to 77.15)	74.63 ± 25.45 (73.57 to 75.69)	84.56 ± 25.62 (83.50 to 85.62)	81.42 ± 24.80 (80.39 to 82.46)

Abbreviations: T − 24: two years before therapy; T − 12: one year before therapy; T + 12: one year after therapy; T + 24: two years after therapy; Post hoc analysis: Recovery: T − 24 vs. T − 12, *p* < 0.0001; T − 24 vs. T + 12 *p* < 0.0001; T − 24 vs. T + 24, *p* < 0.0001; T − 12 vs. T + 12, *p* < 0.0001; T − 12 vs. T + 24, *p* < 0.0001; T + 12 vs. T + 24, *p* = 0.57; BMI: T − 24 vs. T − 12, *p* < 0.0001; T − 24 vs. T + 12, *p* < 0.0001; T − 24 vs. T + 24, *p* < 0.0001; T − 12 vs. T + 12, *p* < 0.0001; T − 12 vs. T + 24, *p* < 0.0001; T + 12 vs. T + 24, *p* = 0.09; BMI z-score: T − 24 vs. T − 12, *p* = 0.15; T − 24 vs. T + 12 *p* < 0.0001; T − 24 vs. T + 24, *p* < 0.0001; T − 12 vs. T + 12, *p* < 0.0001; T − 12 vs. T + 24, *p* < 0.0001; T + 12 vs. T + 24, *p* = 0.95; ppFEV_1_: T − 24 vs. T − 12, *p* < 0.0001; T − 24 vs. T + 12, *p* < 0.0001; T − 24 vs. T + 24, *p* < 0.0001; T − 12 vs. T + 12, *p* < 0.0001; T − 12 vs. T + 24, *p* < 0.0001; T + 12 vs. T + 24, *p* < 0.0001.

**Table 3:** Nutrition and ppFEV_1_ data by genotype.

Parameter	F/F T − 12 Mean ± SD (95%CI)	F/F T + 12 Mean ± SD (95%CI)	F/F T + 24 Mean ± SD (95%CI)	F/Other T − 12 Mean ± SD (95%CI)	F/Other T + 12 Mean ± SD (95%CI)	F/Other T + 24 Mean ± SD (95%CI)
BMI (kg/m^2^) (n = 1093 vs. n = 579)	21.60 ± 2.79 (21.37 to 21.83)	22.59 ± 2.92 (22.35 to 22.83)	22.59 ± 3.00 (22.35 to 22.84)	21.93 ± 3.16 (21.74 to 22.12)	23.02 ± 3.26 (22.83 to 23.22)	23.11 ± 3.36 (22.91 to 23.31)
BMI z-score (n = 369 vs. n = 236)	−0.36 ± 1.06 (−0.49 to −0.22)	−0.17 ± 1.02 (−0.30 to −0.04)	−0.13 ± 1.05 (−0.26 to 0.009)	−0.32 ± 1.06 (−0.43 to −0.22)	−0.09 ± 0.99 (−0.19 to 0.009)	−0.12 ± 0.99 (−0.23 to −0.02)
ppFEV_1_ (%) (n = 1462 vs. n = 814)	76.02 ± 26.00 (74.21 to 77.83)	85.70 ± 25.80 (83.91 to 87.49)	82.69 ± 24.80 (80.96 to 84.42)	73.84 ± 25.19 (72.53 to 75.15)	83.90 ± 25.54 (82.59 to 85.22)	80.72 ± 24.78 (79.44 to 82.00)

Abbreviations: T − 12: one year before therapy; T + 12: one year after therapy; T + 24: two years after therapy; Post hoc analysis: BMI—F/F (T − 12) vs. F/Other (T − 12), *p* = 0.03; F/F (T + 12) vs. F/Other (T + 12), *p* = 0.005; F/F (T + 24) vs. F/Other (T + 24), *p* = 0.002; Trend analysis: BMI—F/F (T − 12) vs. F/F (T + 12), *p* < 0.0001; F/F (T − 12) vs. F/F (T + 24), *p* < 0.0001; F/F (T + 12) vs. F/F (T + 24), *p* = 0.92; F/Other (T − 12) vs. F/Other (T + 12), *p* < 0.0001; F/Other (T − 12) vs. F/Other (T + 24), *p* < 0.0001; F/Other (T + 12) vs. F/Other (T + 24), *p* = 0.03; BMI z-score—F/F (T − 12) vs. F/F (T + 12), *p* < 0.0001; F/F (T − 12) vs. F/F (T + 24), *p* < 0.0001; F/F (T + 12) vs. F/F (T + 24), *p* = 0.09; F/Other (T − 12) vs. F/Other (T + 12), *p* < 0.0001; F/Other (T − 12) vs. F/Other (T + 24), *p* < 0.0001; F/Other (T + 12) vs. F/Other (T + 24), *p* = 0.17; ppFEV_1_—F/F (T − 12) vs. F/F (T + 12), *p* < 0.0001; F/F (T − 12) vs. F/F (T + 24), *p* < 0.0001; F/F (T + 12) vs. F/F (T + 24), *p* < 0.0001; F/Other (T − 12) vs. F/Other (T + 12), *p* < 0.0001; F/Other (T − 12) vs. F/Other (T + 24), *p* < 0.0001; F/Other (T + 12) vs. F/Other (T + 24), *p* < 0.0001.

**Table 4:** Nutrition and ppFEV_1_ data by sex.

Parameter	Female T − 12 Mean ± SD (95%CI)	Female T + 12 Mean ± SD (95%CI)	Female T + 24 Mean ± SD (95%CI)	Male T − 12 Mean ± SD (95%CI)	Male T + 12 Mean ± SD (95%CI)	Male T + 24 Mean ± SD (95%CI)
BMI (kg/m^2^) (n = 799 vs. n = 873)	21.25 ± 3.11 (21.04 to 21.47)	22.21 ± 3.25 (21.98 to 22.43)	22.26 ± 3.37 (22.02 to 22.50)	22.33 ± 2.89 (22.13 to 22.52)	23.48 ± 2.93 (23.28 to 23.68)	23.54 ± 3.00 (23.34 to 23.75)
BMI z-score (n = 316 vs. n = 289)	−0.26 ± 1.01 (−0.37 to −0.15)	−0.06 ± 0.90 (−0.16 to 0.04)	−0.02 ± 0.88 (−0.12 to 0.08)	−0.41 ± 1.11 (−0.54 to −0.28)	−0.19 ± 1.10 (−0.32 to −0.06)	−0.24 ± 1.14 (−0.37 to −0.11)
ppFEV_1_ (%) (n = 1115 vs. n = 1161)	74.03 ± 25.61 (72.50 to 75.56)	84.24 ± 26.14 (82.69 to 85.78)	80.91 ± 25.34 (79.41 to 82.41)	75.18 ± 25.38 (73.71 to 76.66)	84.90 ± 25.16 (83.44 to 86.35)	81.91 ± 24.31 (80.50 to 83.32)

Abbreviations: T − 12: one year before therapy; T + 12: one year after therapy; T + 24: two years after therapy; Post hoc analysis: BMI—Female (T − 12) vs. Male (T − 12), *p* < 0.0001; Female (T + 12) vs. Male (T + 12), *p* < 0.0001; Female (T + 24) vs. Male (T + 24), *p* < 0.0001. Trend analysis: BMI—Female (T − 12) vs. Female (T + 12), *p* < 0.0001; Female (T − 12) vs. Female (T + 24), *p* < 0.0001; Female (T + 12) vs. Female (T + 24), *p* = 0.24; Male (T − 12) vs. Male (T + 12), *p* < 0.0001; Male (T − 12) vs. Male (T + 24), *p* < 0.0001; Male (T + 12) vs. Male (T + 24), *p* = 0.14; BMI z-score—Female (T − 12) vs. Female (T + 12), *p* < 0.0001; Female (T − 12) vs. Female (T + 24), *p* < 0.0001; Female (T + 12) vs. Female (T + 24), *p* = 0.08; Male (T − 12) vs. Male (T + 12), *p* < 0.0001; Male (T − 12) vs. Male (T + 24), *p* < 0.0001; Male (T + 12) vs. Male (T + 24), *p* = 0.08; ppFEV_1_—Female (T − 12) vs. Female (T + 12), *p* < 0.0001; Female (T − 12) vs. Female (T + 24), *p* < 0.0001; Female (T + 12) vs. Female (T + 24), *p* < 0.0001; Male (T − 12) vs. Male (T + 12), *p* < 0.0001; Male (T − 12) vs. Male (T + 24), *p* < 0.0001; Male (T + 12) vs. Male (T + 24), *p* < 0.0001.

**Table 5:** ppFEV_1_ by age.

Parameter	Paediatric T − 12 Mean ± SD (95%CI)	Paediatric T + 12 Mean ± SD (95%CI)	Paediatric T + 24 Mean ± SD (95%CI)	Adult T − 12 Mean ± SD (95%CI)	Adult T + 12 Mean ± SD (95%CI)	Adult T + 24 Mean ± SD (95%CI)
ppFEV_1_ (%) (n = 2276)	92.36 ± 18.96 (90.80 to 93.92)	101.80 ± 17.78 (100.35 to 103.25)	97.90 ± 18.35 (96.38 to 99.42)	67.91 ± 24.73 (66.69 to 69.14)	78.00 ± 25.48 (76.73 to 79.26)	74.99 ± 24.36 (73.78 to 76.21)

Abbreviations: T − 12: one year before therapy; T + 12: one year after therapy; T + 24: two years after therapy; Post hoc analysis: ppFEV_1_—Paediatric (T − 12) vs. Adult (T − 12), *p* < 0.0001; Paediatric (T + 12) vs. Adult (T + 12), *p* < 0.0001; Paediatric (T + 24) vs. Adult (T + 24), *p* < 0.0001; Trend analysis: ppFEV_1_—Paediatric (T − 12) vs. Paediatric (T + 12), *p* < 0.0001; Paediatric (T − 12) vs. Paediatric (T + 24), *p* < 0.0001; Paediatric (T + 12) vs. Paediatric (T + 24), *p* < 0.0001; Adult (T − 12) vs. Adult (T + 12), *p* < 0.0001; Adult (T − 12) vs. Adult (T + 243), *p* < 0.0001; Adult (T + 12) vs. Adult (T + 24), *p* = 0.0001.

**Table 6:** Nutrition and ppFEV_1_ data by absence or presence of CF-related diabetes.

Parameter	Diabetes (Negative) T − 12 Mean ± SD (95%CI)	Diabetes (Negative) T + 12 Mean ± SD (95%CI)	Diabetes (Negative) T + 24 Mean ± SD (95%CI)	Diabetes (Positive) Mean ± SD (95%CI)	Diabetes (Positive) Mean ± SD (95%CI)	Diabetes (Positive) Mean ± SD (95%CI)
BMI (kg/m^2^) (n = 1107 vs. n = 472)	22.03 ± 3.14 (21.85 to 22.22)	23.05 ± 3.25 (22.86 to 23.24)	23.10 ± 3.35 (22.90 to 23.30)	21.39 ± 2.83 (21.13 to 21.65)	22.61 ± 2.91 (22.35 to 22.87)	22.66 ± 3.02 (22.38 to 22.93)
BMI z-score (n = 529 vs. n = 58)	−0.31 ± 1.02 (−0.40 to −0.22)	−0.10 ± 0.98 (−0.18 to −0.02)	−0.09 ± 0.96 (−0.18 to 0.00)	−0.43 ± 0.96 (−0.69 to −0.17)	−0.27 ± 1.20 (−0.59 to 0.04)	−0.35 ± 1.27 (−0.72 to 0.01)
ppFEV_1_ (%) (n = 1635 vs. n = 530))	78.13 ± 24.88 (76.91 to 79.35)	87.85 ± 24.80 (86.64 to 89.06)	84.09 ± 24.17 (82.90 to 85.27)	63.38 ± 24.99 (61.24 to 65.52)	73.91 ± 26.16 (71.67 to 76.15)	72.11 ± 25.42 (69.94 to 74.29)

Abbreviations: T − 12: one year before therapy; T + 12: one year after therapy; T + 24: two years after therapy; Post hoc analysis: BMI–Diabetes (Negative) (T − 12) vs. Diabetes (Positive) (T − 12), *p* < 0.0001; Diabetes (Negative) (T + 12) vs. Diabetes (Positive) (T + 12), *p* = 0.008; Diabetes (Negative) (T + 24) vs. Diabetes (Positive) (T + 24), *p* = 0.01; ppFEV_1_—Diabetes (Negative) (T − 12) vs. Diabetes (Positive) (T − 12), *p* < 0.0001; Diabetes (Negative) (T + 12) vs. Diabetes (Positive) (T + 12), *p* < 0.0001; Diabetes (Negative) (T + 24) vs. Diabetes (Positive) (T + 24), *p* < 0.0001; Trend analysis: BMI—Diabetes (Negative) (T − 12) vs. Diabetes (Negative) (T + 12), *p* < 0.0001; Diabetes (Negative) (T − 12) vs. Diabetes (Negative) (T + 24), *p* < 0.0001; Diabetes (Negative) (T + 12) vs. Diabetes (Negative) (T + 24), *p* = 0.19; Diabetes (Positive) (T − 12) vs. Diabetes (Positive) (T + 12), *p* < 0.0001; Diabetes (Positive) (T − 12) vs. Diabetes (Positive) (T + 24), *p* < 0.0001; Diabetes (Positive) (T + 12) vs. Diabetes (Positive) (T + 24), *p* = 0.40; BMI z-score—Diabetes (Negative) (T − 12) vs. Diabetes (Negative) (T + 12), *p* = 0.0009; Diabetes (Negative) (T − 12) vs. Diabetes (Negative) (T + 24), *p* = 0.0006; Diabetes (Negative) (T + 12) vs. Diabetes (Negative) (T + 24), *p* = 0.88; Diabetes (Positive) (T − 12) vs. Diabetes (Positive) (T + 12), *p* = 0.44; Diabetes (Positive) (T − 12) vs. Diabetes (Positive) (T + 24), *p* = 0.73; Diabetes (Positive) (T + 12) vs. Diabetes (Positive) (T + 24), *p* = 0.74; ppFEV_1_—Diabetes (Negative) (T − 12) vs. Diabetes (Negative) (T + 12), *p* < 0.0001; Diabetes (Negative) (T − 12) vs. Diabetes (Negative) (T + 24), *p* < 0.0001; Diabetes (Negative) (T + 12) vs. Diabetes (Negative) (T + 24), *p* < 0.0001; Diabetes (Positive) (T − 12) vs. Diabetes (Positive) (T + 12), *p* < 0.0001; Diabetes (Positive) (T − 12) vs. Diabetes (Positive) (T + 24), *p* < 0.0001; Diabetes (Positive) (T + 12) vs. Diabetes (Positive) (T + 24), *p* < 0.0001.

**Table 7:** Nutrition and ppFEV_1_ data by absence or presence of chronic infection by *Pseudomonas aeruginosa*.

Parameter	*P. aeruginosa* (Negative) T − 12 Mean ± SD (95%CI)	*P. aeruginosa* (Negative) T + 12 Mean ± SD (95%CI)	*P. aeruginosa* (Negative) T + 24 Mean ± SD (95%CI)	*P. aeruginosa* (Positive) T − 12 Mean ± SD (95%CI)	*P. aeruginosa* (Positive) T + 12 Mean ± SD (95%CI)	*P. aeruginosa* (Positive) T + 24 Mean ± SD (95%CI)
BMI (kg/m^2^) (n = 629 vs. n = 1035)	22.07 ± 3.21 (21.82 to 22.33)	23.04 ± 3.42 (22.77 to 23.31)	22.99 ± 3.51 (22.71 to 23.27)	21.66 ± 2.92 (21.48 to 21.83)	22.78 ± 2.97 (22.60 to 22.96)	22.89 ± 3.07 (22.70 to 23.08)
BMI z-score (n = 396 vs. n = 208)	−0.30 ± 1.07 (−0.41 to −0.19)	−0.09 ± 1.00 (−0.19 to 0.009)	−0.07 ± 0.99 (−0.17 to 0.02)	−0.42 ± 1.02 (−0.56 to −0.28)	−0.19 ± 1.01 (−0.33 to −0.05)	−0.22 ± 1.07 (−0.36 to −0.07)
ppFEV_1_ (%) (n = 1025 vs. n = 1241)	82.72 ± 24.14 (81.23 to 84.21)	91.97 ± 23.57 (90.52 to 93.42)	88.51 ± 23.22 (87.07 to 89.96)	67.99 ± 24.56 (66.61 to 69.36)	78.55 ± 25.60 (77.12 to 79.98)	75.66 ± 24.55 (74.29 to 77.04)

Abbreviations: *P. aeruginosa*: *Pseudomonas aeruginosa*; T − 12: one year before therapy; T + 12: one year after therapy; T + 24: two years after therapy; Post hoc analysis: BMI—*P. aeruginosa* (Negative) (T − 12) vs. *P. aeruginosa* (Positive) (T − 12), *p* = 0.008; *P. aeruginosa* (Negative) (T + 12) vs. *P. aeruginosa* (Positive) (T + 12), *p* = 0.12; *P. aeruginosa* (Negative) (T + 24) vs. *P. aeruginosa* (Positive) (T + 24), *p* = 0.55; ppFEV_1_—*P. aeruginosa* (Negative) (T − 12) vs. *P. aeruginosa* (Positive) (T − 12), *p* < 0.0001; *P. aeruginosa* (Negative) (T + 12) vs. *P. aeruginosa* (Positive) (T + 12), *p* < 0.0001; *P. aeruginosa* (Negative) (T + 24) vs. *P. aeruginosa* (Positive) (T + 24), *p* < 0.0001; Trend analysis: BMI—*P. aeruginosa* (Negative) (T − 12) vs. *P. aeruginosa* (Negative) (T + 12), *p* < 0.0001; *P. aeruginosa* (Negative) (T − 12) vs. *P. aeruginosa* (Negative) (T + 24), *p* < 0.0001; *P. aeruginosa* (Negative) (T + 12) vs. *P. aeruginosa* (Negative) (T + 243), *p* = 0.38; *P. aeruginosa* (Positive) (T − 12) vs. *P. aeruginosa* (Positive) (T + 12), *p* < 0.0001; *P. aeruginosa* (Positive) (T − 12) vs. *P. aeruginosa* (Positive) (T + 24), *p* < 0.0001; *P. aeruginosa* (Positive) (T + 12) vs. *P. aeruginosa* (Positive) (T + 24), *p* = 0.006; BMI z-score—*P. aeruginosa* (Negative) (T − 12) vs. *P. aeruginosa* (Negative) (T + 12), *p* = 0.0009; *P. aeruginosa* (Negative) (T − 12) vs. *P. aeruginosa* (Negative) (T + 24), *p* = 0.0006; *P. aeruginosa* (Negative) (T + 12) vs. *P. aeruginosa* (Negative) (T + 24), *p* = 0.52; *P. aeruginosa* (Positive) (T − 12) vs. *P. aeruginosa* (Positive) (T + 12), *p* < 0.0001; *P. aeruginosa* (Positive) (T − 12) vs. *P. aeruginosa* (Positive) (T + 24), *p* < 0.0001; *P. aeruginosa* (Positive) (T + 12) vs. *P. aeruginosa* (Positive) (T + 24), *p* = 0.39; ppFEV_1_—*P. aeruginosa* (Negative) (T − 12) vs. *P. aeruginosa* (Negative) (T + 12), *p* < 0.0001; *P. aeruginosa* (Negative) (T − 12) vs. *P. aeruginosa* (Negative) (T + 24), *p* < 0.0001; *P. aeruginosa* (Negative) (T + 12) vs. *P. aeruginosa* (Negative) (T + 24), *p* < 0.0001; *P. aeruginosa* (Positive) (T − 12) vs. *P. aeruginosa* (Positive) (T + 12), *p* < 0.0001; *P. aeruginosa* (Positive) (T − 12) vs. *P. aeruginosa* (Positive) (T + 24), *p* < 0.0001; *P. aeruginosa* (Positive) (T + 12) vs. *P. aeruginosa* (Positive) (T + 24), *p* < 0.0001.

**Table 8:** Nutrition and ppFEV_1_ data by severity of lung disease.

Parameter	ppFEV_1_ < 40% T − 12 Mean ± SD (95%CI)	ppFEV_1_ < 40% T + 12 Mean ± SD (95%CI)	ppFEV_1_ < 40% T + 24 Mean ± SD (95%CI)	ppFEV_1_ ≥ 40% T − 12 Mean ± SD (95%CI)	ppFEV_1_ ≥ 40% T + 12 Mean ± SD (95%CI)	ppFEV_1_ ≥ 40% T + 24 Mean ± SD (95%CI)
BMI (kg/m^2^) (n = 147 vs. n = 1452)	20.72 ± 2.83 (20.26 to 21.19)	22.33 ± 2.80 (21.88 to 22.78)	22.29 ± 2.80 (21.83 to 22.75)	21.98 ± 3.00 (21.83 to 22.14)	22.98 ± 3.16 (22.81 to 23.14)	23.04 ± 3.25 (22.87 to 23.21)
BMI z-score (n = 4 vs. n = 578)	−0.80 ± 0.83 (−2.87 to 1.27)	−1.57 ± 2.02 (−4.77 to 1.64)	−1.25 ± 1.99 (−4.42 to 1.92)	−0.32 ± 1.03 (−0.40 to −0.23)	−0.10 ± 0.98 (−0.18 to −0.02)	−0.11 ± 0.98 (−0.19 to −0.02)
ppFEV_1_ (%) (n = 151 vs. n = 2029)	31.38 ± 7.70 (30.07 to 32.70)	37.33 ± 10.07 (35.69 to 38.97)	37.98 ± 8.85 (36.51 to 39.46)	78.35 ± 23.07 (77.33 to 79.36)	88.51 ± 22.61 (87.52 to 89.50)	84.99 ± 22.41 (84.01 to 85.98)

Abbreviations: T − 12: one year before therapy; T + 12: one year after therapy; T + 24: two years after therapy; Post hoc analysis; BMI—ppFEV1 < 40% (T − 12) vs. ppFEV1 ≥ 40% (T − 12), *p* < 0.0001; ppFEV1 < 40% (T + 12) vs. ppFEV1 ≥ 40% (T + 12), *p* = 0.009; ppFEV1 < 40% (T + 24) vs. ppFEV1 ≥ 40% (T + 24), *p* = 0.003; ppFEV_1_—ppFEV1 < 40% (T − 12) vs. ppFEV1 ≥ 40% (T − 12), *p* < 0.0001; ppFEV1 < 40% (T + 12) vs. ppFEV1 ≥ 40% (T + 12), *p* < 0.0001; ppFEV1 < 40% (T + 24) vs. ppFEV1 ≥ 40% (T + 24), *p* < 0.0001; Trend analysis: BMI—ppFEV1 < 40% (T − 12) vs. ppFEV1 < 40% (T + 12), *p* < 0.0001; ppFEV1 < 40% (T − 12) vs. ppFEV1 < 40% (T + 24), *p* < 0.0001; ppFEV1 < 40% (T + 12) vs. ppFEV1 < 40% (T + 24), *p* = 0.74; ppFEV1 ≥ 40% (T − 12) vs. ppFEV1 ≥ 40% (T + 12), *p* < 0.0001; ppFEV1 ≥ 40% (T − 12) vs. ppFEV1 ≥ 40% (T + 24), *p* < 0.0001; ppFEV1 ≥ 40% (T + 12) vs. ppFEV1 ≥ 40% (T + 24), *p* = 0.04; BMI z-score—ppFEV1 < 40% (T − 12) vs. ppFEV1 < 40% (T + 12), *p* = 0.43; ppFEV1 < 40% (T − 12) vs. ppFEV1 < 40% (T + 24), *p* = 0.64; ppFEV1 < 40% (T + 12) vs. ppFEV1 < 40% (T + 24), *p* = 0.80; ppFEV1 ≥ 40% (T − 12) vs. ppFEV1 ≥ 40% (T + 12), *p* = 0.0004; ppFEV1 ≥ 40% (T − 12) vs. ppFEV1 ≥ 40% (T + 24), *p* = 0.0006; ppFEV1 ≥ 40% (T + 12) vs. ppFEV1 ≥ 40% (T + 24), *p* = 0.97; ppFEV_1_—ppFEV1 < 40% (T − 12) vs. ppFEV1 < 40% (T + 12), *p* < 0.0001; ppFEV1 < 40% (T − 12) vs. ppFEV1 < 40% (T + 24), *p* < 0.0001; ppFEV1 < 40% (T + 12) vs. ppFEV1 < 40% (T + 24), *p* = 0.35; ppFEV1 ≥ 40% (T − 12) vs. ppFEV1 ≥ 40% (T + 12), *p* < 0.0001; ppFEV1 ≥ 40% (T − 12) vs. ppFEV1 ≥ 40% (T + 24), *p* < 0.0001; ppFEV1 ≥ 40% (T + 12) vs. ppFEV1 ≥ 40% (T + 24), *p* < 0.0001.

**Table 9:** Changes in complications, lung microbiology, and chronic treatments from one year before to two years after ETI initiation.

Parameter	T − 12 Positive n (%)	T + 12 Positive n (%)	*p* *	T − 12 Positive n (%)	T + 24 Positive n (%)	*p* *	T + 12 Positive n (%)	T + 24 Positive n (%)	*p* *
*Pseudomonas aeruginosa*	824 (40.08)	914 (44.45)	**<0.0001**	801 (39.89)	825 (41.09)	0.25	990 (45.47)	921 (42.31)	**0.0003**
ABPA	79 (3.80)	42 (2.02)	**<0.0001**	79 (3.88)	21 (1.03)	**<0.0001**	42 (1.93)	23 (1.06)	**0.001**
Diabetes	402 (19.62)	392 (19.13)	0.43	391 (19.38)	473 (23.44)	**<0.0001**	412 (19.25)	506 (23.64)	**<0.0001**
Pneumothorax	5 (0.24)	12 (0.58)	0.14	4 (0.20)	3 (0.15)	1.00	12 (0.55)	3 (0.14)	**0.03**
Haemoptysis	150 (7.80)	39 (2.03)	**<0.0001**	146 (7.75)	44 (2.34)	**<0.0001**	37 (1.70)	47 (2.16)	0.24
Malignancy	8 (0.39)	8 (0.39)	1.00	7 (0.35)	10 (0.50)	0.63	8 (0.37)	13 (0.60)	0.36
NaCl	1145 (54.71)	1043 (49.83)	**<0.0001**	1125 (54.98)	995 (48.63)	**<0.0001**	1083 (49.63)	1053 (48.26)	0.07
Antibiotic	1101 (52.55)	960 (45.82)	**<0.0001**	1073 (52.39)	811 (39.60)	**<0.0001**	1005 (46.02)	870 (39.83)	**<0.0001**
BD	1684 (80.46)	1723 (82.32)	0.07	1639 (80.15)	1704 (83.33)	**0.002**	1791 (82.04)	1817 (83.23)	0.13
Oxygen	116 (5.63)	82 (3.98)	**0.0003**	110 (5.46)	76 (3.77)	**0.0009**	86 (3.94)	85 (3.89)	0.91
RhDNase	1269 (60.57)	1198 (57.18)	**0.001**	1236 (60.38)	1086 (53.05)	**<0.0001**	1243 (56.94)	1152 (52.77)	**<0.0001**
AZM	824 (39.35)	574 (27.41)	**<0.0001**	807 (39.42)	467 (22.81)	**<0.0001**	600 (27.47)	499 (22.85)	**<0.0001**
UDCA	910 (43.46)	922 (44.03)	0.47	894 (43.67)	953 (46.56)	**0.0006**	976 (44.69)	1024 (46.89)	**0.001**
Pancreatic	1806 (86.21)	1825 (87.11)	**0.02**	1762 (86.03)	1775 (86.67)	0.11	1892 (86.63)	1891 (86.58)	1.00

Abbreviations: T − 12—one year before therapy; T + 12—one year after therapy; T + 24—two years after therapy; * *p* obtained using McNemar test; ETI—elexacaftor/tezacaftor/ivacaftor; ABPA—allergic bronchopulmonary aspergillosis; NaCl—inhaled continuous hypertonic saline; Antibiotic—inhaled continuous antibiotics; BD—inhaled continuous bronchodilators; RhDNase—inhaled continuous recombinant human deoxyribonuclease; AZM—oral continuous azithromycin; UDCA—oral continuous ursodeoxycholic acid; Pancreatic—oral continuous pancreatic enzymes replacement.

**Table 10:** Changes in liver disease from one year before to two years after ETI initiation.

Parameter	T − 12 n (%)	T + 12 n (%)	*p* *	T − 12 n (%)	T + 24 n (%)	*p* *	T + 12 n (%)	T + 24 n (%)	*p* *
LD without cirrhosis	758 (37.90)	534 (26.70)	**<0.0001**	741 (37.94)	952 (48.74)	**<0.0001**	573 (27.32)	1028 (49.02)	**<0.0001**
LD with cirrhosis	54 (4.59)	38 (3.23)	0.052	45 (4.49)	46 (4.59)	0.74	39 (3.53)	60 (5.43)	0.051

Abbreviations: T − 12—one year before therapy; T + 12—one year after therapy; T + 24—two years after therapy; ETI—elexacaftor/tezacaftor/ivacaftor; LD: liver disease; * *p* obtained using McNemar test.

**Table 11:** Changes in chronic treatments from one year before to two years after ETI initiation in subjects with ppFEV_1_ < 40 at baseline.

Parameter	T − 12 Positive n (%)	T + 12 Positive n (%)	*p* *	T − 12 Positive n (%)	T + 24 Positive n (%)	*p* *	T + 12 Positive n (%)	T + 24 Positive n (%)	*p* *
NaCl	78 (60.00)	54 (41.54)	**0.0005**	77 (62.10)	60 (48.39)	**0.006**	66 (46.15)	67 (46.85)	1.00
Antibiotic	93 (71.54)	95 (73.08)	0.83	90 (72.58)	83 (66.94)	0.25	103 (72.03)	96 (67.13)	0.25
BD	118 (90.77)	106 (81.54)	**0.04**	112 (90.32)	107 (86.29)	0.38	120 (83.92)	125 (87.41)	0.44
Oxygen	46 (37.40)	42 (34.15)	0.56	42 (35.90)	33 (28.21)	0.13	42 (29.37)	40 (27.97)	0.72
RhDNase	72 (55.38)	60 (46.15)	**0.03**	67 (54.03)	54 (43.55)	**0.01**	70 (48.95)	68 (47.55)	0.81
AZM	70 (53.84)	47 (36.15)	**<0.0001**	66 (53.23)	42 (33.87)	**<0.0001**	50 (34.97)	49 (34.27)	1.00
UDCA	72 (55.38)	67 (51.54)	0.42	69 (55.65)	63 (50.81)	0.26	73 (51.03)	72 (50.35)	0.83

Abbreviations: T − 12—one year before therapy; T + 12—one year after therapy; T + 24—two years after therapy; * *p* obtained using McNemar test; ETI—elexacaftor/tezacaftor/ivacaftor; NaCl—inhaled continuous hypertonic saline; Antibiotic—inhaled continuous antibiotics; BD—inhaled continuous bronchodilators; RhDNase—inhaled continuous recombinant human deoxyribonuclease; AZM—oral continuous azithromycin; UDCA—oral continuous ursodeoxycholic acid.

**Table 12:** Changes in chronic treatments from one year before to two years after ETI initiation in subjects with ppFEV_1_ ≥ 40 at baseline.

Parameter	T − 12 Positive n (%)	T + 12 Positive n (%)	*p* *	T − 12 Positive n (%)	T + 24 Positive n (%)	*p* *	T + 12 Positive n (%)	T + 24 Positive n (%)	*p* *
NaCl	1017 (54.04)	946 (50.27)	**0.0006**	998 (54.21)	899 (48.83)	**<0.0001**	977 (49.90)	947 (48.37)	0.053
Antibiotic	966 (51.27)	837 (44.93)	**<0.0001**	942 (51.11)	706 (38.31)	**<0.0001**	873 (44.54)	752 (38.37)	**<0.0001**
BD	1506 (79.98)	1562 (82.95)	**0.005**	1467 (79.68)	1539 (83.60)	**0.0003**	1618 (82.59)	1634 (83.41)	0.32
Oxygen	59 (3.17)	36 (1.94)	**0.002**	57 (3.13)	35 (1.92)	**0.003**	40 (2.04)	38 (1.94)	0.71
RhDNase	1154 (61.25)	1105 (58.65)	**0.02**	1126 (61.13)	1005 (54.56)	**<0.0001**	1140 (58.19)	1056 (53.91)	**<0.0001**
AZM	732 (38.87)	510 (27.08)	**<0.0001**	719 (39.03)	410 (22.26)	**<0.0001**	532 (27.19)	434 (22.14)	**<0.0001**
UDCA	797 (42.33)	812 (43.12)	0.34	782 (42.45)	842 (45.71)	**0.0002**	860 (43.88)	905 (46.17)	**0.0009**

Abbreviations: T − 12—one year before therapy; T + 12—one year after therapy; T + 24—two years after therapy; * *p* obtained using McNemar test; ETI—elexacaftor/tezacaftor/ivacaftor; NaCl—inhaled continuous hypertonic saline; Antibiotic—inhaled continuous antibiotics; BD—inhaled continuous bronchodilators; RhDNase—inhaled continuous recombinant human deoxyribonuclease; AZM—oral continuous azithromycin; UDCA—oral continuous ursodeoxycholic acid.

## Data Availability

The datasets used and analysed during the current study are available from the corresponding author on reasonable request.

## Supplementary Materials

The following supporting information can be downloaded at https://www.mdpi.com/article/10.3390/jcm15072699/s1. Table S1: Nutrition and ppFEV_1_ data by genotype subgroups; Table S2: Nutrition and ppFEV_1_ data by severity of lung disease subgroups.

## Abbreviations

The following abbreviations are used in this manuscript:

CF	Cystic Fibrosis
ETI	Elexacaftor/tezacaftor/ivacaftor
CFTR	Cystic Fibrosis Transmembrane Regulator
pwCF	People with Cystic Fibrosis
ICFR	Italian Cystic Fibrosis Registry
BMI	Body Mass Index
ppFEV_1_	Percent predicted FEV_1_
PEx	Pulmonary exacerbation
ABPA	Allergic Bronchopulmonary Aspergillosis
CFRD	Cystic Fibrosis-Related Diabetes
LD	Liver Disease
*P. aeruginosa*	*Pseudomonas aeruginosa*
BD	Bronchodilator
AZM	Azithromycin
UDCA	Ursodeoxycolic acid
F	F508del
F/G	F/gating
F/MF	F/Minimal function
F/RF	F/Residual function
