# Supplementary material for: Real-World Effectiveness of Elexacaftor/Tezacaftor/Ivacaftor in Cystic Fibrosis: A 24-Month Italian National Registry Study
Source: J Clin Med. 2026 Apr 2;15(7):2699. doi: 10.3390/jcm15072699 (PMC13073555; doi:10.3390/jcm15072699)
Supplement: Supplementary file 1 [file jcm-15-02699-s001.zip › jcm-4185493-supplementary.pdf]

Table S1 – **Nutrition and ppFEV$_1$ data by genotype subgroups**

Parameter	F508del/ Other T-12 Mean±SD (95%CI)	F508del/ Other T+12 Mean±SD (95%CI)	F508del/ Other T+24 Mean±SD (95%CI)	F508del/ Gating T-12 Mean±SD (95%CI)	F508del/ Gating T+12 Mean±SD (95%CI)	F508del/ Gating T+24 Mean±SD (95%CI)	F508del/ Residual Function T-12 Mean±SD (95%CI)	F508del/ Residual Function T+12 Mean±SD (95%CI)	F508del/ Residual Function T+24 Mean±SD (95%CI)	F508del/ Minimal Function T-12 Mean±SD (95%CI)	F508del /Minimal Function T+12 Mean±SD (95%CI)	F508del/ Minimal Function T+24 Mean±SD (95%CI)
BMI (kg/m^2)	22.53±3.90 (21.95 to 23.12)	23.50±4.04 (22.90 to 24.10)	23.56±4.21 (22.93 to 24.20)	23.03±3.52 (21.80 to 24.26)	23.92±3.44 (22.73 to 25.12)	23.92±3.49 (22.69 to 25.14)	23.29±3.70 (22.73 to 23.85)	24.02±3.83 (23.44 to 24.60)	24.14±3.98 (23.52 to 24.75)	21.43±2.66 (21.23 to 21.63)	22.63± 2.80 (22.43 to 22.84)	22.72±2.87 (22.51 to 22.93)
BMI z score	-0.29±0.97 (-0.54 to -0.04)	-0.07±0.92 (-0.30 to 0.17)	-0.12±0.97 (-0.38 to 0.14)	0.08±1.42 (-1.04 to 1.19)	0.01±1.21 (-0.93 to 0.96)	0.13±1.68 (-1.42 to 1.68)	0.004±1.09 (-0.44 to 0.45)	0.17±1.06 (-0.28 to 0.62)	0.30±0.82 (-0.16 to 0.76)	-0.33±1.01 (-0.45 to -0.21)	-0.12±1.00 (-0.24 to -0.002)	-0.14±0.98 (-0.26 to -0.02)
ppFEV$_1$ (%)	73.33±25.29 (70.96 to 75.68)	82.81±26.59 (80.36 to 85.26)	78.88±25.66 (76.39 to 81.16)	67.94±28.31 (58.52 to 77.36)	73.72±29.61 (64.30 to 83.14)	67.90±27.94 (58.96 to 76.84)	79.61± 21.98 (75.79 to 83.43)	85.93±22.33 (82.12 to 89.74)	81.53± 22.14 (77.70 to 85.37)	73.45±25.33 (71.71 to 75.19)	84.62± 25.14 (82.90 to 86.33)	82.11± 24.45 (80.43 to 83.79)

Abbreviations: T-12: one year before therapy; T+12: one year after therapy; T+24: two years after therapy

***Post hoc* analysis**

BMI - F508del/Other (T-12) vs. F508del/Gating (T-12), p=0.47; F508del/Other (T-12) vs. F508del/Residual Function (T-12), p=0.07; F508del/Other (T-12) vs. F508del/Minimal Function (T-12), p=**0.0005**; F508del/Other (T+12) vs. F508del/Gating (T+12), p=0.53; F508del/Other (T+12) vs. F508del/Residual Function (T+12), p=0.22; F508del/Other (T+12) vs. F508del/Minimal Function (T+12), p=**0.007**; F508del/Other (T+24) vs. F508del/Gating (T+24), p=0.61; F508del/Other (T+24) vs. F508del/Residual Function (T+24), p=0.21; F508del/Other (T+24) vs. F508del/Minimal Function (T+24), p=**0.01**

F508del/Gating (T-12) vs. F508del/Residual Function (T-12), p=0.70; F508del/Gating (T-12) vs. F508del/Minimal Function (T-12), p=**0.01**; F508del/Gating (T+12) vs. F508del/Residual Function (T+12), p=0.88; F508del/Gating (T+12) vs. F508del/Minimal Function (T+12), p=0.03; F508del/Gating (T+24) vs. F508del/Residual Function (T+24), p=0.75; F508del/Gating (T+24) vs. F508del/Minimal Function (T+24), p=0.054; F508del/Residual Function (T-12) vs. F508del/Minimal Function (T-12), p**<0.0001**; F508del/Residual Function (T+12) vs. F508del/Minimal Function (T+12), p**<0.0001**; F508del/Residual Function (T+24) vs. F508del/Minimal Function (T+24), p**<0.0001**

BMI z score - F508del/Other (T-12) vs. F508del/Gating (T-12), p=0.53; F508del/Other (T-12) vs. F508del/Residual Function (T-12), p=0.25; F508del/Other (T-12) vs. F508del/Minimal Function (T-12), p=0.77; F508del/Other (T+12) vs. F508del/Gating (T+12), p=0.87; F508del/Other (T+12) vs. F508del/Residual Function (T+12), p=0.36; F508del/Other (T+12) vs. F508del/Minimal Function (T+12), p=0.69; F508del/Other (T+24) vs. F508del/Gating (T+24), p=0.75; F508del/Other (T+24) vs. F508del/Residual Function (T+24), p=0.12; F508del/Other (T+24) vs. F508del/Minimal Function (T+24), p=0.88; F508del/Gating (T-12) vs. F508del/Residual Function (T-12), p=0.90;

F508del/Gating (T-12) vs. F508del/Minimal Function (T-12), p=0.47; F508del/Gating (T+12) vs. F508del/Residual Function (T+12), p=0.77; F508del/Gating (T+12) vs. F508del/Minimal Function (T+12), p=0.79; F508del/Gating (T+24) vs. F508del/Residual Function (T+24), p=0.84; F508del/Gating (T+24) vs. F508del/Minimal Function (T+24), p=0.73 F508del/Residual Function (T-12) vs. F508del/Minimal Function (T-12), p=0.15; F508del/Residual Function (T+12) vs. F508del/Minimal Function (T+12), p=0.22; F508del/Residual Function (T+24) vs. F508del/Minimal Function (T+24), p=0.07

ppFEV$_1$ - F508del/Other (T-12) vs. F508del/Gating (T-12), p=0.27; F508del/Other (T-12) vs. F508del/Residual Function (T-12), p=**0.006**; F508del/Other (T-12) vs. F508del/Minimal Function (T-12), p=0.94; F508del/Other (T+12) vs. F508del/Gating (T+12), p=0.06; F508del/Other (T+12) vs. F508del/Residual Function (T+12), p=0.18; F508del/Other (T+12) vs. F508del/Minimal Function (T+12), p=0.24; F508del/Other (T+24) vs. F508del/Gating (T+24), p=**0.02**; F508del/Other (T+24) vs. F508del/Residual Function (T+24), p=0.23; F508del/Other (T+24) vs. F508del/Minimal Function (T+24), p=**0.02**; F508del/Gating (T-12) vs. F508del/Residual Function (T-12), p=**0.02**; B (T-12) vs. F508del/Minimal Function (T-12), p=0.25; F508del/Gating (T+12) vs. F508del/Residual Function (T+12), p=**0.02**; F508del/Gating (T+12) vs. F508del/Minimal Function (T+12), p=**0.02**; F508del/Gating (T+24) vs. F508del/Residual Function (T+24), p=**0.006**; F508del/Gating (T+24) vs. F508del/Minimal Function (T+24), p=**0.002**; F508del/Residual Function (T+12) vs. F508del/Minimal Function (T+12), p=**0.004**; F508del/Residual Function (T+12) vs. F508del/Minimal Function (T+12), p=0.54; F508del/Residual Function (T+24) vs. D (T+24), p=0.79

Trend analysis

BMI - F508del/Other (T-12) vs. F508del/Other (T+12), p**<0.0001**; F508del/Other (T-12) vs. F508del/Other (T+24), p**<0.0001**; F508del/Other (T+12) vs. F508del/Other (T+24), p=0.54; F508del/Gating (T-12) vs. F508del/Gating (T+12), p=**0.02**; F508del/Gating (T-12) vs. F508del/Gating (T+24), p=**0.001**; F508del/Gating (T+12) vs. F508del/Gating (T+24), p=0.99; F508del/Residual Function (T-12) vs. F508del/Residual Function (T+12), p**<0.0001**; F508del/Residual Function (T-12) vs. F508del/Residual Function (T+24), p**<0.0001**; F508del/Residual Function (T+12) vs. F508del/Residual Function (T+24), p=0.29; F508del/Minimal Function (T-12) vs. F508del/Minimal Function (T+12), p**<0.0001**; F508del/Minimal Function (T-12) vs. F508del/Minimal Function (+24T), p**<0.0001**; F508del/Minimal Function (T+12) vs. F508del/Minimal Function (T+24), p=**0.048**

BMI z score - F508del/Other (T-12) vs. F508del/Other (T+12), p=0.19; F508del/Other (T-12) vs. F508del/Other (T+24), p=0.35; F508del/Other (T+12) vs. F508del/Other (T+24), p=0.76; F508del/Gating (T-12) vs. F508del/Gating (T+12), p=0.06; F508del/Gating (T-12) vs. F508del/Gating (+24T), p=0.97; F508del/Gating (T+12) vs. F508del/Gating (+24T), p=0.93; F508del/Residual Function (T-12) vs. F508del/Residual Function (T+12), p=0.32; F508del/Residual Function (T-12) vs. F508del/Residual Function (+24T), p=0.33; F508del/Residual Function (T+12) vs. F508del/Residual Function (T+24), p=0.33; F508del/Minimal Function (T-12) vs. F508del/Minimal Function (T+12), p=0.09; F508del/Minimal Function (T-12) vs. F508del/Minimal Function (T+24), p=0.09; F508del/Minimal Function (T+12) vs. F508del/Minimal Function (T+24), p=0.08

ppFEV$_1$ - F508del/Other (T-12) vs. F508del/Other (T+12), p**<0.0001**; F508del/Other (T-12) vs. F508del/Other (T+24), p**<0.0001**; F508del/Other (T+12) vs. F508del/Other (T+24), p**<0.0001**; F508del/Gating (T-12) vs. F508del/Gating (T+12), p=**0.008**; F508del/Gating (T-12) vs. F508del/Gating (T+24), p=0.98; F508del/Gating (T+12) vs. F508del/Gating (T+24), p**<0.0001**; F508del/Residual Function (T-12) vs. F508del/Residual Function (T+12), p**<0.0001**; F508del/Residual Function (T-12) vs. F508del/Residual Function (T+24), p=**0.03**; F508del/Residual Function (T+12) vs. F508del/Residual Function (T+24), p**<0.0001**; F508del/Minimal Function (T-12) vs. F508del/Minimal Function (T+12), p**<0.0001**; F508del/Minimal Function (T-12) vs. F508del/Minimal Function (+24T), p**<0.0001**; F508del/Minimal Function (T+12) vs. F508del/Minimal Function (T+24), p**<0.0001**

Table S2 – Nutrition and ppFEV$_1$ data by severity of lung disease subgroups

Parameter	T-12 ppFEV$_1$<40% Mean±SD (95%CI)	T+12 ppFEV$_1$<40% Mean±SD (95%CI)	T+24 ppFEV$_1$<40% Mean±SD (95%CI)	T-12 40≤ppFEV$_1$<70 %; Mean±SD (95%CI)	T+12 40≤ppFEV$_1$<70 %; Mean±SD (95%CI)	T+24 40≤ppFEV$_1$<70 %; Mean±SD (95%CI)	T-12 ppFEV$_1$≥70% Mean±SD (95%CI)	T+12 ppFEV$_1$≥70% Mean±SD (95%CI)	T+24 ppFEV$_1$≥70% Mean±SD (95%CI)
BMI (kg/m^2)	20.72±2.83 (20.26 to 21.19)	22.33±2.80 (21.88 to 22.78)	22.29±2.80 (21.83 to 22.75)	21.55±3.14 (21.28 to 21.81)	22.83±3.29 (22.56 to 23.12)	22.95±3.43 (22.66 to 23.24)	22.24±2.88 (20.05 to 22.43)	23.06±3.08 (22.86 to 23.27)	23.11±3.15 (22.90 to 23.31)
BMI z score	-0.80±0.83 (-2.87 to 1.27)	-1.57±2.02 (-4.77 to 1.64)	-1.25±1.99 (-4.42 to 1.92)	-0.79±1.16 (-1.13 to -0.44)	-0.40±1.09 (-0.72 to -0.09)	-0.44±1.08 (-0.75 to -0.13)	-0.28±1.01 (-0.36 to -0.19)	-0.08±0.97 (-0.16 to 0.006)	-0.07±0.97 (-0.16 to 0.01)
ppFEV$_1$ (%)	31.38±7.70 (30.07 to 32.70)	37.33±10.07 (35.69 to 38.97)	37.98±8.85 (36.51 to 39.46)	51.78±11.77 (50.79 to 52.76)	61.99±13.64 (60.87 to 63.11)	60.06±13.65 (58.93 to 61.19)	89.07±17.14 (88.18 to 89.96)	99.26±15.59 (98.45 to 100.06)	95.10±16.51 (94.24 to 95.96)

Abbreviations: T-12: one year before therapy; T+12: one year after therapy; T+24: two years after therapy

***Post hoc* analysis**

BMI - ppFEV$_1$<40% (T-12) vs. 40≤ppFEV$_1$<70% (T-12), p=**0.002**; ppFEV$_1$<40% (T-12) vs. ppFEV$_1$≥70 (T-12), p**<0.0001**; 40≤ppFEV$_1$<70% (T-12) vs. ppFEV$_1$≥70% (T-12), p**<0.0001**; ppFEV$_1$<40% (T+12) vs. 40≤ppFEV$_1$<70% (T+12), p=**0.007**; ppFEV$_1$<40% (T+12) vs. ppFEV$_1$≥70% (T+12), p=**0.004**; 40≤ppFEV$_1$<70% (T+12) vs. ppFEV$_1$≥70% (T+12), p=0.19; ppFEV$_1$<40% (T+24) vs. 40≤ppFEV$_1$<70% (T+24), p=**0.02**; ppFEV$_1$<40% (T+24) vs. ppFEV$_1$≥70% (T+24), p=**0.001**; 40≤ppFEV$_1$<70% (T+24) vs. ppFEV$_1$≥70% (T+24), p=0.39

BMI z score - ppFEV$_1$<40% (T-12) vs. 40≤ppFEV$_1$<70% (T-12), p=0.97; ppFEV$_1$<40% (T-12) vs. ppFEV$_1$≥70% (T-12), p=0.18; 40≤ppFEV$_1$<70% (T-12) vs. ppFEV$_1$≥70% (T-12), p=**0.005**; ppFEV$_1$<40% (T+12) vs. 40≤ppFEV$_1$<70% (T+12), p=0.19; ppFEV$_1$<40% (T+12) vs. ppFEV$_1$≥70% (T+12), p=0.09; 40≤ppFEV$_1$<70% (T+12) vs. ppFEV$_1$≥70% (T+12), p=**0.048**; ppFEV$_1$<40% (T+24) vs. 40≤ppFEV$_1$<70% (T+24), p=0.35; ppFEV$_1$<40% (T+24) vs. ppFEV$_1$≥70% (T+24), p=0.17; 40≤ppFEV$_1$<70% (T+24) vs. ppFEV$_1$≥70% (T+24), p=**0.03**

ppFEV$_1$ - ppFEV$_1$<40% (T-12) vs. 40≤ppFEV$_1$<70% (T-12), p**<0.0001**; ppFEV$_1$<40% (T-12) vs. ppFEV$_1$≥70% (T-12), p**<0.0001**; 40≤ppFEV$_1$<70% (T-12) vs. ppFEV$_1$≥70% (T-12), p**<0.0001**; ppFEV$_1$<40% (T+12) vs. 40≤ppFEV$_1$<70% (T+12), p**<0.0001**; ppFEV$_1$<40% (T+12) vs. ppFEV$_1$≥70% (T+12), p**<0.0001**; 40≤ppFEV$_1$<70% (T+12) vs. ppFEV$_1$≥70% (T+12), p**<0.0001**; ppFEV$_1$<40% (T+24) vs. 40≤ppFEV$_1$<70% (T+24), p**<0.0001**; ppFEV$_1$<40% (T+24) vs. ppFEV$_1$≥70% (T+24), p**<0.0001**; 40≤ppFEV$_1$<70% (T+24) vs. ppFEV$_1$≥70% (T+24), p**<0.0001**

Trend analysis

BMI - ppFEV$_1$<40% (T-12) vs. ppFEV$_1$<40% (T+12), p**<0.0001**; ppFEV$_1$<40% (T-12) vs. ppFEV$_1$<40% (T+24), p**<0.0001**; ppFEV$_1$<40% (T+12) vs. ppFEV$_1$<40% (T+24), p=0.62; 40≤ppFEV$_1$<70% (T-12) vs. 40≤ppFEV$_1$<70% (T+12), p**<0.0001**; 40≤ppFEV$_1$<70% (T-12) vs. 40≤ppFEV$_1$<70% (T+24), p**<0.0001**; 40≤ppFEV$_1$<70% (T+12) vs. 40≤ppFEV$_1$<70% (T+24), p=**0.03**; ppFEV$_1$≥70% (T-12) vs. ppFEV$_1$≥70% (T+12), p**<0.0001**; ppFEV$_1$≥70% (T-12) vs. ppFEV$_1$≥70% (T+24), p**<0.0001**; ppFEV$_1$≥70% (T+12) vs. ppFEV$_1$≥70% (T+24), p=0.32

BMI z score - ppFEV$_1$<40% (T-12) vs. ppFEV$_1$<40% (T+12), p=0.42; ppFEV$_1$<40% (T-12) vs. ppFEV$_1$<40% (T+24), p=0.63; ppFEV$_1$<40% (T+12) vs. ppFEV$_1$<40% (T+24), p=0.80; 40≤ppFEV$_1$<70% (T-12) vs. 40≤ppFEV$_1$<70% (T+12), p=0.10; 40≤ppFEV$_1$<70% (T-12) vs. 40≤ppFEV$_1$<70% (T+24), p=0.14; 40≤ppFEV$_1$<70% (T+12) vs. 40≤ppFEV$_1$<70% (T+24), p=**0.03**; ppFEV$_1$≥70% (T-12) vs. ppFEV$_1$≥70% (T+12), p=**0.001**; ppFEV$_1$≥70% (T-12) vs. ppFEV$_1$≥70% (T+24), p=**0.001**; ppFEV$_1$≥70% (T+12) vs. ppFEV$_1$≥70% (T+24), p=0.96

$ppFEV_1$ - $ppFEV_1$<40% (T-12) vs. $ppFEV_1$<40% (T+12), p**<0.0001**; $ppFEV_1$<40% (T-12) vs. $ppFEV_1$<40% (T+24), p**<0.0001**; $ppFEV_1$<40% (T+12) vs. $ppFEV_1$<40% (T+24), p=0.34; 40≤$ppFEV_1$<70% (T-12) vs. 40≤$ppFEV_1$<70% (T+12), p**<0.0001**; 40≤$ppFEV_1$<70% (T-12) vs. 40≤$ppFEV_1$<70% (T+24), p**<0.0001**; 40≤$ppFEV_1$<70% (T+12) vs. 40≤$ppFEV_1$<70% (T+24), p**<0.0001**; $ppFEV_1$≥70% (T-12) vs. $ppFEV_1$≥70% (T+12), p**<0.0001**; $ppFEV_1$≥70% (T-12) vs. $ppFEV_1$≥70% (T+24), p**<0.0001**; $ppFEV_1$≥70% (T+12) vs. $ppFEV_1$≥70% (T+24), p**<0.0001**
